# Playing-related musculoskeletal disorders among Chinese conservatoire piano students: prevalence, risk factors and preventive interventions

**DOI:** 10.3389/fpsyg.2024.1386661

**Published:** 2024-10-14

**Authors:** Luqian Zhao, Yafei Wang, Zheren Zhang

**Affiliations:** ^1^School of Music, University of Leeds, Leeds, United Kingdom; ^2^School of Arts and Philosophy, Shinawatra University, Pathum Thani, Thailand

**Keywords:** piano, musculoskeletal disorders, playing-related injuries, conservatoire students, prevalence, risk factors, preventive interventions

## Abstract

**Introduction:**

Both professional musicians and conservatoire students are at significant risk of developing playing-related musculoskeletal disorders (PRMDs) during their career life. With the growing number of students pursuing a conservatory degree and graduating from music conservatory in China, the aims of this study were: (1) to identify the nature of PRMD and explore the prevalence of PRMD in Chinese conservatoire students; (2) to determine the relevant risk factors with the presence of PRMD among Chinese conservatoire students; and (3) to suggest preventive interventions for young pianists at their early career stage.

**Methods:**

A self-reported online survey study was conducted among 363 Chinese conservatoire students who majored in piano performance.

**Results:**

Of all respondents, 82.6% reported having had at least one PRMD. The wrist was proved to be the most affected body site, followed by the shoulder, finger and arm. Respondents who experienced PRMD reported “pain,” “fatigue,” and “stiffness” as the most frequent symptoms. The main risk factors associated with PRMD included gender, years of playing experiences, practice hours, warm-up habits before practice, and break-taking during practice.

**Discussion:**

Female students, those with longer year of playing experience, those who practice longer hours, those who do not warm up before practice, and those who do not take breaks during practice were found to have more PRMD symptoms and higher level of severity. This study highlights the need to increase awareness of PRMD among conservatoire students and to understand the occurrence of PRMD; it is helpful for young pianists to prevent severe musculoskeletal disorders and implement preventive measures at early career stages. Further studies are suggested to follow up on music students who have had at least one PRMD at different stages of professional musical training.

## 1 Introduction

Throughout the career life of a musician, it is common to experience mild aches and pains during playing and after playing. These symptoms, known as playing-related musculoskeletal symptoms (PRMS), are generally milder than playing-related musculoskeletal disorders (PRMD) (Brandfonbrener, 2003; Ranelli et al., 2011). PRMDs are a common issue among musicians during their whole career life (Brandfonbrener, 2003; Zaza, 1998), including pains and symptoms that affect the ability to play the instrument as usual (Zaza and Farewell, 1997). For professional musicians, these occupational-related symptoms can result in a loss of performing opportunity, leading to decreased income, social stigma, and psychological and emotional challenges associated with being unable to engage in music-making (Guptill, 2011).

The high prevalence of PRMDs among professional musicians has been attributed to chronic musculoskeletal conditions that were overlooked and improperly treated during their younger years (Bejjani et al., 1984; Savvidou and Stanek, 2019). Most existing studies regarding PRMDs have focused primarily on professional musicians (Ackermann et al., 2011; Hoppmann and Patrone, 1989; Steinmetz et al., 2015), often neglecting the early pain experiences of musicians in their younger ages. This gap in the literature is significant because conservatoire students, who are at the early stages of their professional careers, are also susceptible to PRMD. More recent studies have started to highlight the equivalent prevalence of PRMD among conservatoire students (Brandfonbrener, 2009; Cruder, 2020; Steemers et al., 2020). Understanding and addressing these early pain experiences is crucial for preventing chronic issues in later professional life as a musician.

Research has provided insight into the nature and prevalence of PRMD in musicians across different disciplines. Performing arts and medical research approach the study of PRMDs with different focuses and measurements. Performing arts literature tends to categorize PRMDs based on instruments and/or playing positions, often using less standardized definitions (e.g., Dawson, 2002; Sakai, 2002; Taddey, 1992). This can lead to a wider range of disorders being included and thus a higher reported prevalence rate, especially when mild symptoms and pains are classified as disorders. Occupational medicine literature, though more limited, groups PRMDs by medically confirmed diseases that meet the established diagnosis standards in the late 1980s (Wilke et al., 2011). Consequently, some unconfirmed disorders might be overlooked in these studies.

Existing literature shows varied results regarding the prevalence rates of PRMD due to differences in research methods (e.g., questionnaires, interviews, or physical examination), measurements (i.e., number of musicians experiencing PRMDs at a specific point in time, or over a specific period such as a month or a year), instruments and small sample sizes. Reported prevalence rates of PRMD range from 40 to 60% among professional musicians (Zaza and Farewell, 1997; Fry, 1986), 9 to 90% among tertiary music students (Fry, 1987; Zetterberg et al., 1998), and 20 to 70% among children learning instrumental music (Brown, 1997; Grieco et al., 1989; Roset-Llobet et al., 2000). In the systematic review conducted by Zaza (1998), the prevalence rate of PRMD among professional musicians ranged from 39 to 87%.

The prevalence of PRMD among keyboard players was significantly higher compared to that of percussionists (Sandell et al., 2009). However, within the group of keyboard players, pianists exhibited the lowest prevalence of PRMD (Ranelli et al., 2011). Among tertiary music students, those playing strings, woodwind, brass, and keyboard were reported to have a high prevalence of PRMD (Zetterberg et al., 1998; Pratt et al., 1992).

In medicine literature (Biro et al., 1983; Buescher, 2007; Caldron et al., 1986; Hochberg et al., 1988; Konczak and Abbruzzese, 2013; Larsson et al., 1993; Newmark and Hochberg, 1987; Sataloff et al., 1991; Zeuner and Molloy, 2008), four main PRMDs were identified: overuse syndrome, temporomandibular joint disorders, focal motor dystopias and joint hypermobility. Overuse syndrome is defined as painful conditions induced by long and hard use of a limb beyond biological tolerance (Fry, 1986; Fry, 1993). It is often used generically to include conditions such as tendinitis, tenosynovitis, dystonia, and related conditions that are not currently defined as specific disorders (e.g., Altenmüller, 2003; Altenmüller et al., 2012; Fry, 1986; Marsden, 1991). As a result, the diagnosis of symptoms is generally based on the doctor’s own clinical experience (Bejjani et al., 1996). These conditions are also related to the instrument played or the position of playing, which aligns with the findings in performing arts literature.

Although much research has been done to explore the prevalence of PRMD in different instrument players and factors associated with musicians’ occupational-related injuries (e.g., Bejjani et al., 1993; Papandreou and Vervainioti, 2010), limited is focused on the specific group of conservatoire piano students. While a professional career is not necessarily the goal of every conservatoire student, avoiding PRMD should be a priority at any level of professional piano training. The risk factors contributing to PRMD in musicians are multifactorial, including individual factors (such as age, gender, previous injuries, anthropometrical characteristics of the musician, and psychological distress) (Ranelli et al., 2011; Cruder et al., 2021; Pfalzer and Walker, 2001; Warrington et al., 2002; Yoshie et al., 2009), instrument-related factors (such as weight of the instrument, techniques, and training of playing) (Mikimoto et al., 2004), playing conditions (e.g., time spent playing the instrument) (Fry, 1987; Fotiadis et al., 2013; Ranelli et al., 2011; Robitaille et al., 2018), and the interactions between these factors.

To simultaneously address the gap in existing literature and a lack of evidence for the population of Chinese pianists, this study aims: (1) To ascertain the prevalence and symptoms of PRMD in Chinese conservatoire students majoring in piano performance; (2) To determine any risk factors of the appearance of PRMD. Based on existing literatures, we hypothesized that age, gender, years of playing, practice hours, practice habits and strategies would be associated with PRMD; (3) To suggest interventive preventions for conservatoire piano students.

## 2 Materials and methods

This study adopted a self-reported online questionnaire. This questionnaire was structured based on an existing survey by Ling et al. (2018); therefore, the validity of the questionnaire was already established.

Participants were recruited through contacts with conservatoires in China. Recruitment was done in three ways. Firstly, an invitation email to participate in this study was sent to their teaching staffs of piano studies, and then the link to questionnaire was shared with their students. Secondly, online advertisements via social media platforms were published. Thirdly, we utilized the word-of-mouth strategy by asking for referrals from people who have participated in previous surveys.

This study aims to recruit piano students because the research aim is to examine the prevalence of PRMD among Chinese Conservatoire Piano students. Therefore, Chinese students majoring in piano performance at undergraduate or postgraduate level are considered.

At the beginning of the questionnaire, a brief introduction of the study, including its purpose and procedure, was given. Furthermore, the level of confidentiality, which would be upheld throughout the project, and the voluntary nature of participation were demonstrated. Participants were informed that personal information would be kept anonymized and confidential. Due to the voluntary nature of the self-reported online questionnaire, informed consent was obtained before participants answered the questionnaire by having them tick a consent box. Contact details of the two researchers were provided at the end of the questionnaire if participants have any further enquiries.

### 2.1 Questionnaire design and procedure

The questionnaire was structured into three sections, and it took approximately 7 to 10 min to complete. The design of this questionnaire was informed by the existing survey used by Ling et al. (2018), as their survey was designed specifically for pianists and closely aligned with the research objectives of the current study. The reliability and validity of the questionnaire were already established in their studies. The first section collected respondents’ demographic information (gender, age, age started playing the piano, number of years learning the piano). In the second section, participants were asked about their practice habits, such as years of playing experiences, weekly practice hours, break-taking during practice and warm-up exercises. In the third section, participants were required to indicate their experiences of PRMD during their entire piano training. Only participants who had experienced any symptoms before were included. The list of injury locations included finger, wrist, arm, neck, shoulder and back. A blank space was also provided for participants to identify discomfort in any other parts of the body. For each discomfort, they were asked to describe the discomfort in the body parts that they have experienced using checkboxes (allowing for multiple selections).

Participants were further asked to rate the level of discomfort they have experienced. The level of discomfort was used to categorize the severity of PRMD on a scale of 0 to 7 (as shown in Table 1). Levels 0 to 2 were regarded as relatively mild discomfort, and thus, only those who had rated severity at level 3 and above were considered experiencing PRMD. According to Ling et al. (2018), respondents who rated pain at levels 0 to 2 were not considered as having PRMD. Thus, respondents declared a PRMD at level 2 and below were excluded from being classified as having PRMD in the analysis.

**Table 1:** Level of severity of signs and symptoms of pain among pianists.

Level	Signs and symptoms
0	No pain during and after playing the piano.
1	Feeling tired during and after playing the piano, but no pain or other symptoms.
2	Pain occurs while playing the piano, or for a short period of time (< 2 days) after playing the piano. You are still able to play the piano normally.
3	Pain occurs while playing the piano and persists for a longer period (>2 days) after playing the piano. However, playing is not yet restricted.
4	Pain progresses. You have to change playing techniques and reduce playing time. Pain resolves after the alteration.
5	Pain occurs once you start to play the piano. Changing techniques and shortening playing time do not relieve pain. Some daily activities are affected.
6	Pain persists even when you are not playing the piano. Many daily activities are affected. You have to stop playing completely until recovery.
7	Pain persists. No recovery. You are not able to play the piano anymore.

The questionnaire was translated and adapted in Chinese. To ensure that the adapted version was valid for the current context and to test the validity of translation, a pilot study was conducted among a convenience sample of conservatoire students. Their feedback was incorporated into the final design of the questionnaire used in this study. Feedback included the need to provide an approximate length of time required to complete the questionnaire. A copy of the final questionnaire is available in the Supplementary material.

### 2.2 Data analysis

All the data was analyzed using SPSS 26.0. Descriptive statistics are presented for the prevalence rates of PRMD among Chinese piano students, with the prevalence rate calculated as a percentage of reported PRMD of the whole sample. To determine the relationship between the occurrence of PRMD and several variables, analyses of variances, Chi-square and regression analyses were conducted. These analyses were adopted based on their suitability for identifying statistical differences between groups and for exploring associations between variables. In the following sections, the results quantifying the effects of gender, years of playing experience, practice hours, the habit of warm up before practice and break-taking during practice are described. A significance level of *p* < 0.05 was used for reporting.

## 3 Results

The total number of participants was 363 piano performing students drawn from five Chinese conservatoires, including the Central Conservatory of Music, Shanghai Conservatory of Music, Sichuan Conservatory of Music, Zhejiang Conservatory of Music and Xinghai Conservatory of Music. The average age of the sample was 22.3 (min-max = 18–27; *SD* = 2.2). More respondents identified their gender as female (*n* = 241, 66.4%), and other responses were male (*n* = 122, 33.6%). In general, participants had an average of 16 years of playing experience on the piano (min-max = 8–22; *SD* = 2.24). The average age at which they started learning the piano was 8.24 (min-max = 4–13; *SD* = 1.69). The weekly hours spent practicing the piano were reported as 1-5 h (*n* = 30, 8.3%), 6-10 h (*n* = 21, 5.7%), 11-15 h (*n* = 22, 6.1%), 16-20 h (*n* = 71, 19.6%), 21-25 h (*n* = 93, 25.6%), 26-30 h (*n* = 80, 22%), and 30+ hours (*n* = 46, 12.7%).

**Figure 1:**
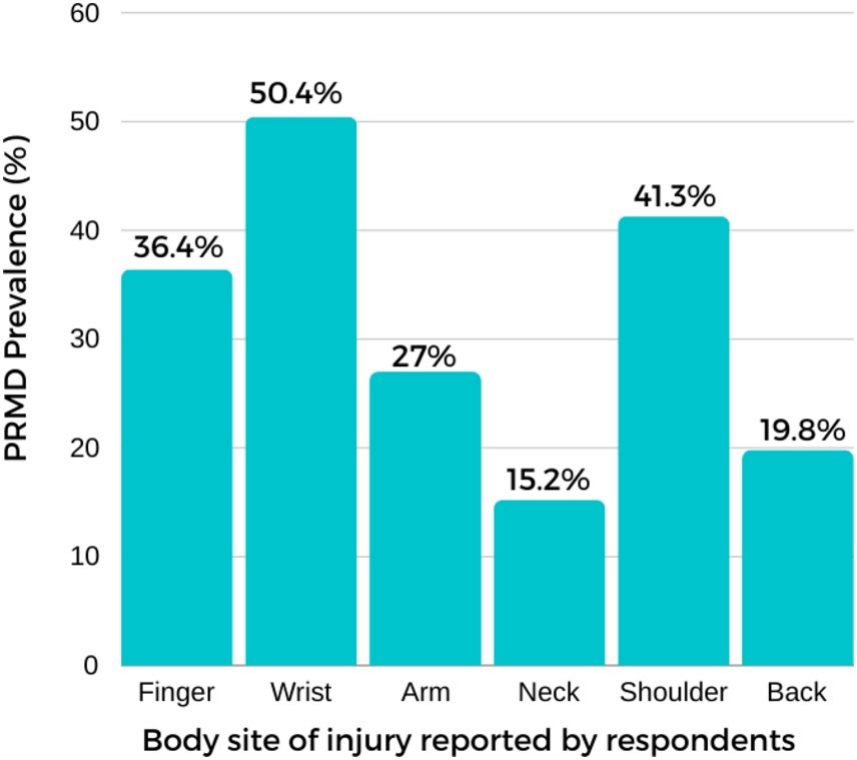
The prevalence of PRMD by reported body site.

**Figure 2:**
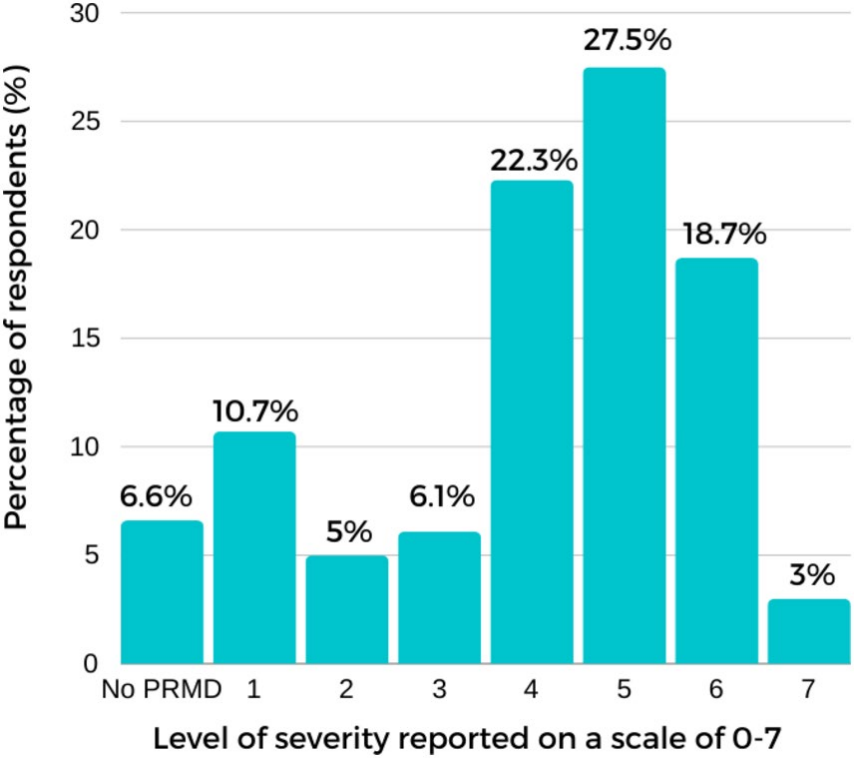
The percentage of respondents for each level of severity reported (on a scale of 0–7).

Of all respondents, 82.6% (300/363) reported having at least one PRMD. The prevalence figures reported in this study refer to lifetime PRMDs experienced by respondents during their entire piano-playing experiences. Participants also reported having experienced multiple signs and symptoms and in more than one body site. The reported symptoms were associated with the upper body. The wrist (*n* = 183, 50.4%) was found to be the most affected body site, followed by the shoulder (*n* = 150, 41.3%), finger (*n* = 132, 36.4%), arm (*n* = 98, 27%), back (*n* = 72, 19.8%) and neck (*n* = 55, 15.2%). Pain (*n* = 211, 58.1%), fatigue (*n* = 188, 51.8%), and stiffness (*n* = 153, 42.15%) were the most cited symptoms. The average severity rating was highest in the shoulder (M = 5.33; *SD* = 3), wrist (M = 4.69; *SD* = 3.49), finger (M = 4.37; *SD* = 3.84) and arm (M = 3.92; *SD* = 3.42). Figure 1 provides the prevalence of PRMD by body site. Figure 2 presents the distribution of severity ratings, including data for those at severity level 1–2 who were excluded in further analyses, for descriptive purposes. Most respondents (77.6%) reported having symptoms, and among these respondents, 68.6% (*n* = 249) reported having had severe signs and symptoms rated between levels 4 and 6 (see Figure 2 for the distribution of the maximum severity reported).

**Figure 3:**
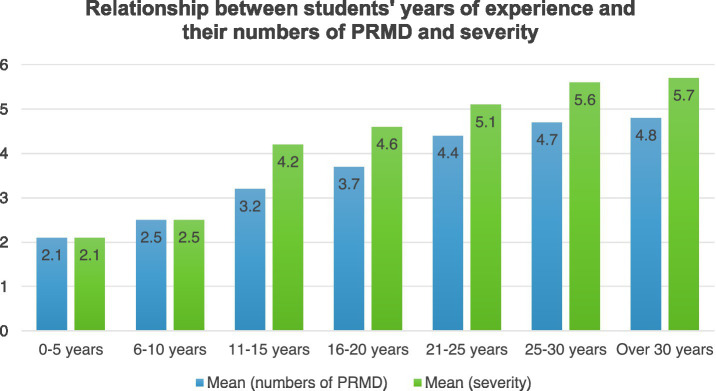
Relationship between students’ years of experience and their numbers of PRMD and severity.

This study identifies significant associations between PRMD prevalence and various factors, however definitive causals conclusions regarding these relationships were not drawn. The results have shown that there was a statistically significant difference between females and males. Females (M = 3.7, SD = 0.96) were more likely to report more PRMD than males (M = 2.9, SD = 1.17). In addition, females (M = 4.8, SD = 2.24) reported a significantly higher level of severity than males (M = 4.2, SD = 2.62). The year of playing experiences of piano students were found to be significantly related to their prevalence and maximum severity of PRMD. There was potentially a complex relationship between the prevalence of PRMD and years of playing experience (as shown in Figure 3). To test the relationships, linear regressions were carried out between years of playing experience and the maximum severity of injuries. A statistically significant positive linear relationship between the maximum severity of injuries and years of playing experience was found (*B* = 0.71, *p* = 0.03*), suggesting that longer years of playing experience were associated with higher severity.

**Figure 4:**
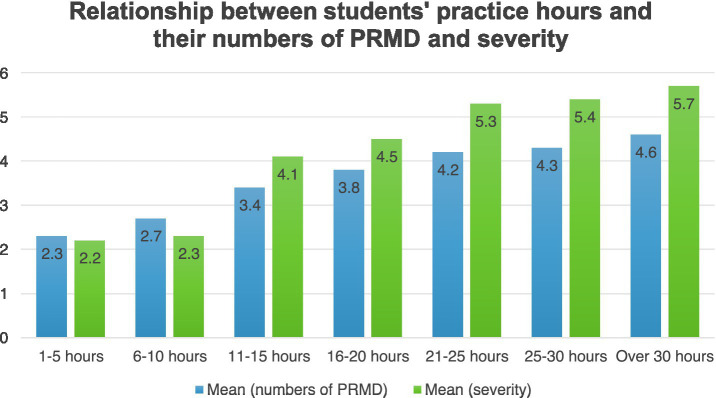
Relationship between students’ practice hours and their numbers of PRMD and severity.

Practice hours of the students were highly correlated to their PRMD prevalence (r = 0.101 [−0.006, −0.126], *p* = 0.013*) and maximum severity of PRMD (*r* = 0.096 [−0.126, −0.113], *p* = 0.021*). As shown in Figure 4, the relationship between practice hours and the severity of PRMD was complex. In order to further explore relationships between students’ practice hours per week and their prevalence and severity of PRMD, a series of multiple linear regressions were conducted. A positive linear relationship between the maximum severity of injuries and practice hours was found and the result was significant (*B* = 1.35, *p* = 0.00*), suggesting that overall longer practice hour was associated with injuries of a higher level of severity.

There were significant associations between students’ practice habits and their PRMD prevalence and severity. Participants were asked to indicate whether they had the habit of warming up before practice and taking breaks during practice. According to their responses, the group that has the habit of warm up before practice showed lower prevalence and severity of PRMD than the other group that has no habit of warm up (results are presented in Table 2). Furthermore, there was a statistically significant difference in PRMD, and severity based on the habit of break- taking during practice: one group that reported the habit of taking break during practice and the other had no habit of taking break during practice. In general, the latter group showed higher prevalence of PRMD and higher severity level of PRMD (results are presented in Table 3).

**Table 2:** Students’ ANOVA test and effect size for the who has the habit of warm up before practice and who has no habit of warm up before practice.

	Group 1: who has the habit of warm up before practice		Group 2: who has no habit of warm up before practice		*t*	*p*
	M	SD	M	SD		
Numbers of PRMD	3.7	1.3	4.3	1.9	56.32	0.001
Severity level	4.5	2.7	5.2	2.3	52.67	0.001

**Table 3:** Students’ ANOVA test and effect size for the who take break during practice and who does not take break during practice.

	Group 1: who take break during practice		Group 2: who does not take break during practice		*t*	*p*
	M	SD	M	SD		
Numbers of PRMD	3.9	1.2	4.7	3.3	58.32	0.005
Severity level	4.4	1.9	5.3	2.2	22.18	0.017

## 4 Discussion

This study aimed to (1) ascertain the prevalence of PRMD in Chinese conservatoire piano students; (2) determine any predicting risk factors associated with PRMD, and (3) evaluate relevant preventive interventions. One advantage of this study is its inclusion of a substantial sample size of Chinese conservatoire students majoring in piano performance.

Of the 363 participants, 82.64% reported having had at least one PRMD during their entire piano-playing experiences, a figure slightly lower than that reported in recent studies (e.g., Savvidou and Stanek, 2019). Although this study has limitations regarding the validity of the severity data, since respondents may have rated injuries that occurred many years ago, introducing potential recall bias, findings are consistent with comparable studies which have reported PRMD prevalence rates among musicians of between 39 to 87% (Zaza, 1998; Baadjou et al., 2016; Berque et al., 2016). The results also align with piano-specific PRMD rates previously reported by Sandell et al. (2009). A majority of participants had experienced severe signs and symptoms: 71.5% reported at least one PRMD rated 4 and higher.

The most common body site of injuries in this study was found to be wrist (including all severity level 0 to 7). In general, disorders associated with the wrist have been reported to be common among instrument players (Brandfonbrener, 2000; Fry, 1986; Hochberg et al., 1983; Grieco et al., 1989; Sandell et al., 2009; Ranelli et al., 2011). Disorders associated with the wrist have also been proposed to be the most reported problems among pianists, as a result of overuse or misuse (Grieco et al., 1989; Ranelli et al., 2011; Shoup, 1995). Furthermore, shoulder, finger, arm and neck were reported to be the most common affected body sites among piano students, which confirms previous findings (Barr et al., 2005; Buckle and Devereux, 2002; Rigg et al., 2003; Roset-Llobet et al., 2000; Siivola et al., 2004; Zaza, 1995). Previous performing arts literatures that categorize PRMD based on playing position and postures, PRMD associated with the upper extremities (hand, arm and shoulder) and neck were reported to be the most common injury body sites among musicians (e.g., Dawson, 1988; Dawson, 2002; Farias et al., 2002).

Gender was identified as one of the risk factors in this study, echoing to previous studies. Gender has been widely investigated as an individual condition factor that may associate with PRMD and the consensus has been reached: female musicians were more likely to report a PRMD than their male counterparts (Pak and Chesky, 2001; Fjellman-Wiklund et al., 2003; Lim and Altenmüller, 2003; Ranelli et al., 2011). In addition, female musicians reported higher severity level than males, and the effect is statistically significant.

Unlike the individual condition factors and instrument-related factors which are less modifiable, many factors associated with PRMD have been found to be related to the musician’s playing behaviors that are adjustable. Two types of playing behaviors were found to contribute to a higher risk of PRMD: misuse and overuse. Misuse includes inappropriate use of the body, which is in relation to practice habits, including playing techniques, movement techniques and postures (Lippmann, 1991). As instruments have their own shapes and weights, wrong positions and body postures can lead to injuries (Brandfonbrener, 2000). Similarly, Guptill and Zaza (2010) argue that all instrument players need to maintain the natural curvatures of the spine (both in sitting and standing) during playing; otherwise, it can lead to PRMD. For instance, the common slouching of shoulders and the hyperextension in the lower spinal of musicians during playing can cause unnecessary tension and pain in the lower and mid-back among musicians (Guptill and Zaza, 2010; Harreby et al., 1995). Also, Lippmann (1991) suggests that the PRMD of pianists were related to misuse of the body and improper practicing habits, including motor skills and movement techniques. For instance, the extreme ranges of movement during playing (Fry, 1986) and wrist postures working against gravitational force (Allsop and Ackland, 2010) have been evidenced to be misuse factors that can lead to overuse syndrome.

The other type of playing behavior that can lead to PRMD is overuse, which includes repetitive movements (Rozmaryn, 1993), long hours of practice and playing, long years of playing experiences, and a sudden increase in practice intensity. Performing and practicing instruments pose significant musculoskeletal pressure on musicians (Kok et al., 2016; Quarrier, 1993), as they often involve intensive repetitive and striking motions that are primary sources of PRMD (Lederman and Calabrese, 1986). Similarly, Guptill and Zaza (2010) argue that the use of repetitive moves during extended hours of practice could be an important risk factor of PRMD. Overuse occurs when muscles, tendons, ligaments or fascia are stressed beyond the individual’s biological limits (Dawson, 2008; Philipson et al., 1990). For pianists, overuse is common, since the fundamental skills in playing the piano are motor skills, which involve enormous movements of hands, fingers, wrists and arms and lead to overuse (Sakai, 2002). Brandfonbrener (2000) criticized repetitive techniques involved in complex repertoires and the needed force applied to keys to cause overuse problems among pianists. Lim and Altenmüller (2003) argued that heavy workloads and non-flexible work schedules were responsible for overuse among musicians. The results of this study confirmed that longer years of playing experience and longer practice hours can contribute to PRMD rate and severity.

The group of participants with the habit of warm-up before practice showed lower PRMD frequency and severity than the group of no warm-up habit. Having warmups before practice can be helpful for pianists to reduce PRMD. Before playing, both musical and physical warm-ups are needed. The importance of musical warm-up that aims to prepare the body and mind for performance has been noted by musicians, and some musical warm-up strategies have been developed and deployed. Slow and comfortable playing can help pianists to prepare themselves musically before performance. It may include starting with moderately paced scales and arpeggios or easy repertoires (Guptill and Zaza, 2010), which, on the one hand, can help pianists get into music quickly and, on the other hand, can prepare the relevant muscles for continued playing. Meanwhile, physical warm-ups such as body stretching can also be helpful, as they can enhance muscle flexibility and benefit quick-speed movements by improving the strength of the musculotendinous unit (Page, 2012). Specific warm-up exercise of stretching is involved in different muscles and body sites. To reduce the risk of overuse syndrome of hands or wrists, Guptill and Zaza (2010) encouraged pianists to turn their palms upward and extend their wrists and fingers.

The risk of overuse syndrome can also be reduced through necessary breaks. It has been found in this study that participants who take breaks during practice showed lower prevalence rate of PRMD and lower severity than those who do not have the habit of break-taking. Taking regular breaks can be beneficial to reduce the occurrence of PRMD. Guptill and Zaza (2010) argued that two types of breaks are needed: micro-breaks during practicing and breaks away from playing. Micro-breaks during playing can be a stop for several seconds or counting rests (*ibid*), which allow for muscle recovery. Breaks away from playing involves longer breaks after a long time of playing. Although many teachers advocate a five-minute break for every thirty-minute practice (Tubiana and Amadio, 2000), no specific studies investigating the time and frequency a musician need to reduce the risk for playing-related injuries. Pianists need to determine the time and frequency of breaks based on their own conditions.

Smart practice techniques can help to reduce the overuse syndrome through reduced practicing hours and repetitions. Less effective practice techniques, for instance, returning to the beginning of the section when a mistake is made and repeating the same section all over again (Renwick and Mcpherson, 2002; Williamon and Valentine, 2000). As a result, smart practice techniques that avoid unnecessary work could be essential to prevent overuse syndrome of hands or wrists for pianists. First, modern technology (e.g., recording applications) can be used to identify errors during practice. After identifying the error, the pianist should practice the error areas slowly and repeatedly rather than returning to bar 1 and practicing the whole repertoire. Secondly, incorporating cognitive practice without the instrument (such as practicing fingering on the table) into daily practice routines can be an effective strategy for reducing the force applied to the keys during piano performance. Other useful cognitive practice techniques include getting familiarized with the structure, phrasing, and interpretation of the repertoire through listening to recordings before playing, visualizing successful performances by other musicians, and imagining the process of playing through the piece mentally (Guptill and Zaza, 2010). These strategies can be helpful in enhancing efficiency and reducing repetitions (Bandura, 1986). Thirdly, developing a structured practice plan helps pianists to manage their practice hours effectively and avoid sudden increases in practice time and frequency that could lead to the occurrence of overuse syndrome.

**Figure 5:**
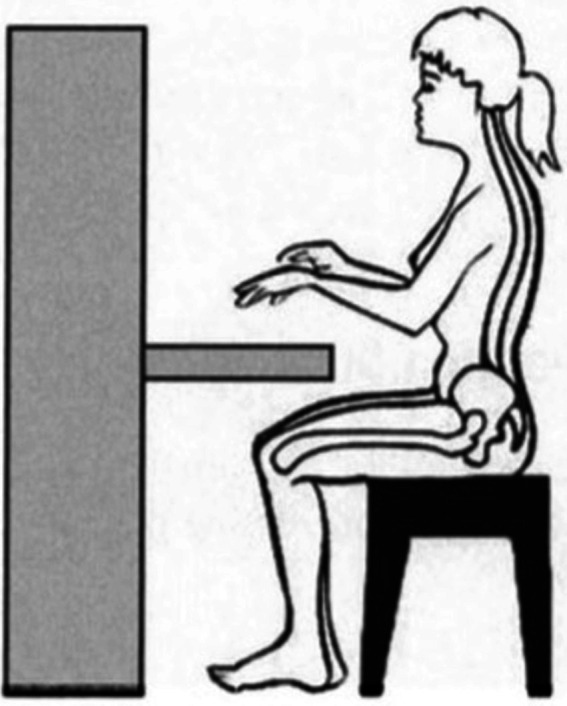
Proper sitting position for piano-playing recommended by Guptill and Zaza (2010, p.30). Adapted from Adalbert (2000).

It is important for musicians, including pianists, to adopt correct postures and playing positions since wrong postures can cause unnecessary tension for muscles and thus lead to a higher risk of playing-related disorders. For pianists, the proper sitting position is shown in Figure 5, typically involving the maintenance of normal curves of the spine when sitting at the piano (Guptill and Zaza, 2010). According to Guptill and Zaza (2010), the recommended piano-playing position is designed to minimise strain on various body sites including hands, wrists and arms. This recommended position facilitates easy and unrestricted movements of the hands and wrists, thereby reducing the risk of playing-related injuries, including overuse syndrome in these body sites.

**Figure 6:**
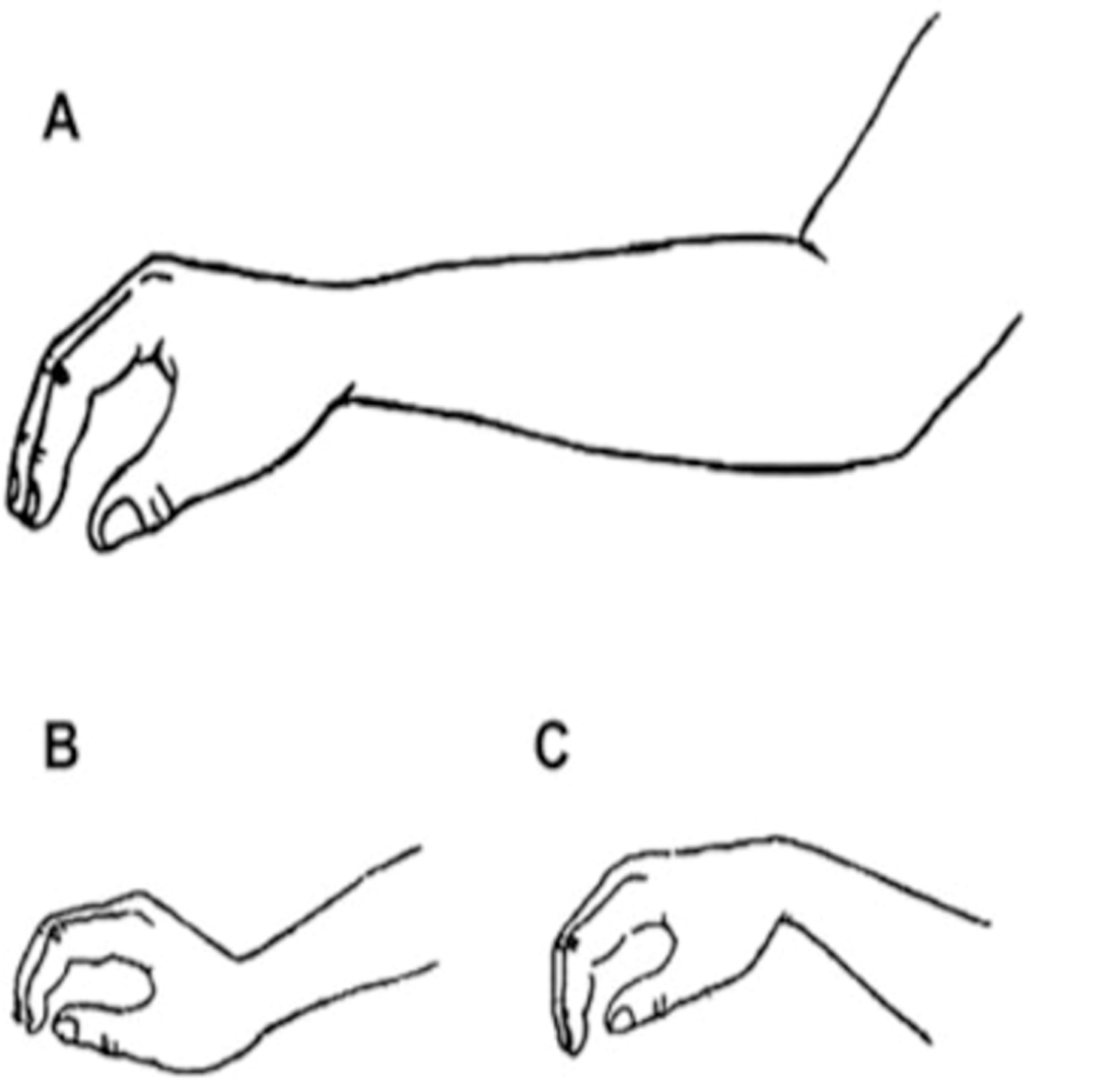
The proper and wrong positions of the wrist during piano-playing suggested by Oikawa et al. (2011). Adapted from (Mikimoto et al., 2004).

The position of the wrist during piano playing is crucial for the overall well-being of the pianist. Oikawa et al. (2011) highlight the significance of minimizing forearm burden to decrease the likelihood of related disorders. Recommendation for pianists could include maintaining a neutral wrist position during playing (see Figure 6). Incorrect position of wrists (such as those shown in Figures 6B,C) may cause an additional burden for the forearm and wrists, as they require extra muscle activities of the wrist’s extensors and flexors (Oikawa et al., 2011). Adhering to proper wrist positioning is essential for pianists to reduce the risk of musculoskeletal issues and promote a healthier playing experience for pianists.

## 5 Conclusion

In summary, this study highlighted a high prevalence of PRMDs among Chinese conservatoire piano students, with 82.64% of participants reporting at least one PRMD. The wrist was identified as the most influenced body site of injury, followed by the shoulder, finger, and arm, with pain, fatigue, and stiffness was found to be the most frequently cited symptoms. Key risk factors contributing to the development and severity of PRMDs included gender, years of playing experience, practice hours, and practice habits such as warming up and taking breaks. Female students, those with longer playing experiences, those practicing for extended hours, and those neglecting warm-ups and breaks were more likely to suffer from PRMDs.

By focusing on the specific population of conservatoire students majoring in piano performance, the research contributes to a more nuanced understanding of the potential challenges faced by piano students in their career life. These insights could potentially inform preventive interventions and strategies aimed at mitigating the risk of PRMD among pianists. The results and findings of this study confirm and build upon existing knowledge regarding PRMD among piano students, suggesting the need for extended research to include professional pianists and longitudinal follow-up studies on music students experiencing PRMDs. Moreover, this study adds to the growing body of literature on PRMD in musicians with a different sample and highlights the importance of addressing these issues to support the physical health and overall well-being of pianists at their early musical career stages.

Despite the valuable insights this study provides into the prevalence and risk factors of PRMDs among Chinese conservatoire piano students, several limitations and challenges must be acknowledged. First, this study relied on self-reported data through an online questionnaire, which may introduce recall bias, leading to insufficient reporting. Participants were asked to recall and rate the severity of injuries that may have occurred many years ago, which could affect the accuracy of the responses. The collection of past events of injury may vary between individuals, and the severity of past symptoms might be over- or under-estimated. This limitation highlights the need for objective measures of PRMD severity, such as clinical assessments or longitudinal data collection. This study employed a cross-sectional design where the data was collected at one point of time. While this approach is useful for identifying the prevalence of PRMDs, it fails to capture changes in PRMD symptoms over time or the progression of disorders. A longitudinal study could make the tracking of PRMD development possible, offering more comprehensive insights into the nature and course of these disorders. These limitations may limit the generalizability of the findings to students from other music schools, or musicians who engage with instruments beyond just the piano. Further research should explore PRMDs across a broader range of musical disciplines and geographical regions to understand the wider applicability of these findings. Additionally, the sampling method might lead to potential sampling bias. The sample might overrepresent individuals who were more engaged with social media or those who have stronger connections with the researchers’ networks. Although the sample size (*n* = 363) is substantial for understanding the prevalence of PRMD among Chinese conservatoire piano students, the recruitment approach may limit the generalizability of the findings to the broader population of musicians. Future studies could aim to employ randomized or stratified sampling techniques to ensure greater representativeness of the sample. Lastly, this study overlooked the importance of other factors influencing the occurrence of PRMD, such as playing posture, psychological stress, and access to ergonomics education for understanding the issue more comprehensively.

## Data Availability

The raw data supporting the conclusions of this article will be made available by the authors, without undue reservation.
