# Supplementary material for: Playing-related musculoskeletal disorders among Chinese conservatoire piano students: prevalence, risk factors and preventive interventions
Source: Front Psychol. 2024 Oct 14;15:1386661. doi: 10.3389/fpsyg.2024.1386661 (PMC11513592; doi:10.3389/fpsyg.2024.1386661)
Supplement: Supplementary file 1 [file Data_Sheet_1.docx]

Chinese Conservatoire Piano Students’ PRMD Survey

*You are being invited to participate in a study which aims to have a better understanding of the prevalence of playing-related injuries and pains during the piano playing among Chinese musicians.*

*The questionnaire is for all conservatoire students who are aged 18 and above and are currently studying piano performance at Chinese conservatoires.*

*Your participation in this study is entirely voluntary and you have the right to withdraw at any point before your response is submitted. By submitting the questionnaire, you are giving the consent to be involved in this study and happy for us to collect your data.*

*Your participation in this study will remain confidential, and only anonymized data will be published. We will minimize any risks by removing all the identifiable information when making the results publicly available.*

Section A: Background

1. Gender:

Female

Male

1. Age:
2. At what age did you begin playing the piano:
3. How long do you learn the instrument?

0-5 years

6-10 years

11-15 years

16-20 years

21-25 years

26-30 years

Over 30 years

Please specify the exact duration:

Section B: Piano Practice Habits

1. Approximately how many hours per day do you usually practice playing piano?
2. Do you do physical warm-up exercises before practice?

Yes

No

1. Do you usually take a break during practice?

Yes

No

Section C: Experiences of Playing-related musculoskeletal disorders

1. Have you experienced any discomfort which affects your piano playing? The discomfort could be in any form as described in Table 1 below, or any other symptoms that affect your piano playing.

Table 1 Description of Types of Symptoms

| Types of discomfort | Description |
| --- | --- |
| Pain | A feeling of physical suffering caused by injury or illness |
| Fatigue | Extreme tiredness |
| Stiffness | Not moving or bending easily, feeling inflexible |
| Numbness | Lack of sensation |
| Pins& needles | Tingling sensation, like being poked by the sharp point of an object |
| Swelling | A part of your body which has become larger and rounder due to illness or injury |
| Spasm | Sudden, uncontrollable, and often painful tightening of music |

1. Please describe the discomfort in the body parts that you have experienced. You can choose more than 1 option from the terms provided in the Table 1. You can also write your own answer if the terms provided do not fit your answer.

| Finger | Wrist | Arm | Neck | Shoulder | Back |
| --- | --- | --- | --- | --- | --- |
| Pain | Pain | Pain | Pain | Pain | Pain |
| Fatigue | Fatigue | Fatigue | Fatigue | Fatigue | Fatigue |
| Stiffness | Stiffness | Stiffness | Stiffness | Stiffness | Stiffness |
| Numbness | Numbness | Numbness | Numbness | Numbness | Numbness |
| Swelling | Swelling | Swelling | Swelling | Swelling | Swelling |
| Spasm | Spasm | Spasm | Spasm | Spasm | Spasm |
| Pins& needles sensation | Pins& needles sensation | Pins& needles sensation | Pins& needles sensation | Pins& needles sensation | Pins& needles sensation |
| Other: | Other: | Other: | Other: | Other: | Other: |

Other parts of the body. Please specify:

Pain

Fatigue

Stiffness

Numbness

Swelling

Spasm

Pins& needles sensation

Other. Please specify:

Referring to your answer to the above question, what is the level of discomfort? Please refer to the pain grading level guidance provided in Table 2.

| Finger | Wrist | Arm | Neck | Shoulder | Back |
| --- | --- | --- | --- | --- | --- |
| Level 0 | Level 0 | Level 0 | Level 0 | Level 0 | Level 0 |
| Level 1 | Level 1 | Level 1 | Level 1 | Level 1 | Level 1 |
| Level 2 | Level 2 | Level 2 | Level 2 | Level 2 | Level 2 |
| Level 3 | Level 3 | Level 3 | Level 3 | Level 3 | Level 3 |
| Level 4 | Level 4 | Level 4 | Level 4 | Level 4 | Level 4 |
| Level 5 | Level 5 | Level 5 | Level 5 | Level 5 | Level 5 |
| Level 6 | Level 6 | Level 6 | Level 6 | Level 6 | Level 6 |
| Level 7 | Level 7 | Level 7 | Level 7 | Level 7 | Level 7 |

Other parts of body. Please specify:

Level 0

Level 1

Level 2

Level 3

Level 4

Level 5

Level 6

Level 7

Table 2 Level of Pain in pianists

| Level | Signs and Symptoms |
| --- | --- |
| 0 | No pain during and after playing piano |
| 1 | Feeling tired during and after playing piano, but no pain or other symptoms |
| 2 | Pain occurs while playing piano, or for a short period of time (< 2 days) after playing piano. The individual is able to play normally |
| 3 | Pain occurs while playing piano and persists for a longer period (>2 days) after class. However, playing piano is not yet restricted |
| 4 | Pain progresses. The individual has to change playing techniques and reduce playing time. Pain resolves after the alteration |
| 5 | Pain occurs once the individual starts to play piano. Changing techniques and shortening playing time do not relieve pain. Some daily activities are affected. |
| 6 | Pain persists even when the individual does not play piano. Many daily activities are affected. The individual has to stop playing piano completely until recovery |
| 7 | Pain persists. No recovery. The individual is not able to play piano anymore. |
